# Relevance of the Isoflavone Absorption and Testicular Function: A Systematic Review of Preclinical Evidence

**DOI:** 10.1155/2021/8853172

**Published:** 2021-02-12

**Authors:** Amanda Alves Lozi, Sérgio Luis Pinto da Matta, Mariáurea Matias Sarandy, Fabiana Cristina Silveira Alves de Melo, Diane Costa Araujo, Rômulo Dias Novaes, Reggiani Vilela Gonçalves

**Affiliations:** ^1^Department of General Biology, Federal University of Viçosa, Viçosa, Minas Gerais, Brazil; ^2^Department of Animal Biology, Federal University of Viçosa, Viçosa, Minas Gerais, Brazil; ^3^Department of Structural Biology, Federal University of Alfenas, Alfenas, Minas Gerais, Brazil

## Abstract

Isoflavone is a phytoestrogen found in different types of food that can act as endocrine disrupters leading to testicular dysfunction. Currently, fragmented data on the action of this compound in the testicles make it difficult to assess its effects to define a safe dose. Thus, we systematically reviewed the preclinical evidence of the impact of isoflavone on testicular function. We also determined which form (aglycones or glycosylated) was the most used, which allowed us to understand the main biological processes involved in testicular function after isoflavone exposure. This systematic review was carried out according to the PRISMA guidelines using a structured search on the biomedical databases MEDLINE (PubMed), Scopus, and Web of Science, recovering and analyzing 22 original studies. The bias analysis and the quality of the studies were assessed by the criteria described in the risk of bias tool developed by SYRCLE (Systematic Review Centre for Laboratory Animal Experimentation). The aglycones and glycosylated isoflavones proved to be harmful to the reproductive health, and the glycosylates at doses of 50, 100, 146, 200, 300, 500, and 600 mg/kg, in addition to 190 and 1000 mg/L, appear to be even more harmful. The main testicular pathologies resulting from the use of isoflavones are associated with Leydig cells resulting from changes in molecular functions and cellular components. The most used isoflavone to evaluate testicular changes was the genistein/daidzein conjugate. The consumption of high doses of isoflavones promotes changes in the functioning of Leydig cells, inducing testicular changes and leading to infertility in murine models.

## 1. Introduction

Phytoestrogens, especially soy isoflavones, are natural nonsteroidal compounds of plant origin found mainly in the Fabaceae family [1]. The chemical structure and function of isoflavones are similar to estradiol and, therefore, their therapeutic effect has been studied in a variety of estrogen-dependent diseases such as menopause, cardiovascular disease, and osteoporosis [1]. Isoflavones such as genistein, daidzein, formononetin, biochanin A, and equol can be found in different types of food such as leguminous (especially soybean), whole grains, and some seeds used in the human diet [2]. Many kinds of food have different types of conjugated isoflavones in their composition, being one of the main sources of exposure to this compound [2]. However, with the discovery of the effects of isoflavones on the body, it became possible to consume isolated isoflavones produced in the laboratory [2].

When isoflavones are absorbed, they can bind to specific estrogen receptors [3] and act as female hormones, mainly altering the levels of luteinizing hormone (LH), follicle stimulating hormone (FSH), and testosterone [4, 5]. In females, the isoflavones genistein and daidzein exhibit beneficial effects on the pituitary-ovary axis and on the ovarian cycle in middle-aged rats [5]. Although most studies are focused on the effect of isoflavones on the female reproductive system, due to their benefits in reducing menopause symptoms, there is evidence that male reproduction can also be affected [6–10]. The exposure of male rats to soy isoflavones can be used to treat and prevent prostate cancer [6–8]. These therapeutic properties can justify a worldwide increase in the consumption of foods rich in isoflavones in recent years [9]. However, its availability in each type of food or even within the same group can vary according to the amount and duration of ingestion [2]. On the other hand, there are studies reporting that the consumption of isoflavones can decrease the production of testosterone and the concentration of sperm in humans (8, 10) leading to infertility [11]. Although there are studies related to the action of isoflavones on testicular function (8, 12), fragmented data make it difficult to define the current evidence and prevent the understanding of the main mechanisms involved in the process of exposure to isoflavones. Currently, it is known that isoflavones act as endocrine disrupters leading to testicular dysfunction [8, 12, 13]. Once in the body, isoflavones can bind to *α* and *β* estrogen receptors inducing weak estrogenic effect [14] and agonistic and/or antagonistic effect on endogenous *β*-estrogen through competition for the same androgen receptors [15]. In addition, a direct inhibition possibly occurs in the germ cells or an alteration of the hypothalamic-pituitary axis [8]. The main consequences of these mechanisms activation are morphological changes that can negatively affect the performance of essential processes for the formation of viable male gametes [16, 17].

In fact, although isoflavones are believed to have several effects on testicular function [18], there is still no consensus to define the best dosage, as well as the main biological processes involving the role of this phytoestrogen in the testicles. Therefore, this study evaluated the action of isoflavones on testicular function in murine models and the relevance, either positive or negative, of this compound to sexual function. We also evaluated the methodological quality of current evidence, indicating the main sources of bias. We believe that our findings may increase the knowledge about the action of isoflavones, aglycone or glycosylates, in biological processes that can alter testicular function. The information obtained in this study can indicate which doses are safe, thus avoiding consumption of harmful quantities to male fertility.

## 2. Materials and Methods

### 2.1. Focus Question

The main question to be answered in this systematic review was, what changes occur in the testicles after the absorption of isoflavones? Second, which isoflavone compound, conjugated or isolated, is most consumed? Third, what are the main biological processes involved in the action of isoflavones in the testicles?

### 2.2. Search Strategy

**Figure 1:**
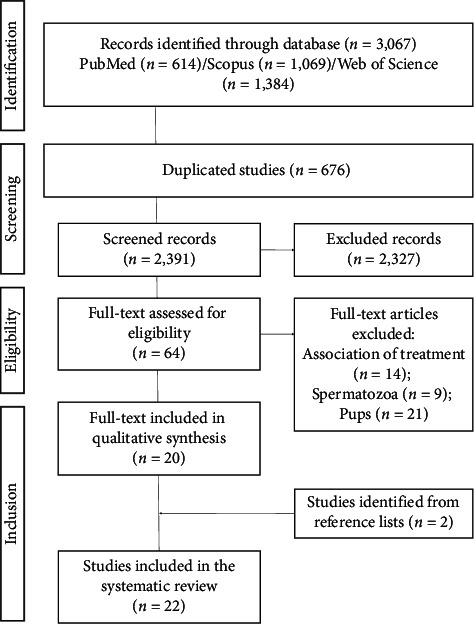
Flow diagram of the systematic review literature search results based on PRISMA statement (Preferred Reporting Items for Systematic Reviews and Meta-Analysis) (http://www.prisma-statement.org/).

All studies included in this systematic review were selected according to the standardized PRISMA guideline (Preferred Reporting Items for Systematic Reviews and Meta-Analysis) [19] (Figure 1). The systematic review was registered at International Prospective Register of Systematic Reviews (PROSPERO) (registration number CRD42020156854) and can be accessed at https://www.crd.york.ac.uk/PROSPERO/. We performed a bibliographic search using the electronic databases PubMed/MEDLINE (https://www.ncbi.nlm.nih.gov/pubmed), Scopus (https://www.scopus.com/home.uri), and Web of Science (https://www-periodicos-capes-gov-br.ez35.periodicos.capes.gov.br) on October 24th, 2019, at 12:57 pm. The search strategy was divided into two steps: (1) direct search in electronic databases and (2) indirect screening of reference lists from all studies identified in the direct search.

The keywords used as search filters were organized in three groups: animal models, testis, and phytoestrogens. Indexed studies were initially retrieved from search filters developed for PubMed by combining keywords and standardized MeSH descriptors (Medical Subject Headings, http://www.ncbi.nlm.nih.gov/mesh). To detect all in vivo preclinical studies, a standardized animal filter was applied [20]. The command [TIAB] (Title and Abstract) was also applied to identify recently published records still in the indexing process. The same research descriptors used for testis and phytoestrogens were structured according to specific search algorithms required in Scopus and Web of Science databases. The Scopus animal filter (keyword-animal (limited to)) was used in this database, and another animal filter was created for Web of Science. In addition, no language limits were applied in the primary search. All studies published up to October 2019 were included in the systematic review. The search strategy is detailed in the supplementary materials (Table S1).

### 2.3. Selection Criteria

Following the PICOS strategy, the studies that met the criteria were included in this systematic review. Two reviewers (AAL and DCA) conducted the literature search, removed duplicate articles, and screened titles and abstracts regarding the eligibility criteria. After the initial screening, full text articles of potentially relevant studies were independently assessed for eligibility by two reviewers (AAL and DCA). The kappa test was used for selection and data extraction (kappa = 0.878). Selections were compared and inconsistencies were resolved in consultation with two other reviewers (RVG and MMS). All studies that evaluated the biological processes of action of the isoflavones in the testicles of murine models were included in our research. Studies with androgens, antagonistic hormones, or evaluation of the action of other compounds, such as pesticides, fungicides, antiandrogens, vitamins, and radiation were excluded. Studies that looked for diseases in other organs, during pregnancy, tumors, females, epididymis-only, sperm, and pups were not included in this systematic review. Only *in vitro*, *in silico*, and *ex vivo* studies on nonrodent animal and studies analyzing vascular disorders were excluded from this review. Secondary studies (literature reviews, letters to the editor, case studies, comments, and editorials) were also excluded.

After screening, all relevant studies were retrieved in full text and selected by eligibility. Eligibility was reviewed by the researchers, and disagreements were resolved by consensus. The reference list of each included study was reviewed and manually tracked for additional articles [21]. If any document was added, its reference list was also revised by the end of this cycle. The results were subjected to enrichment analysis at Enrichr online tool [22, 23].

### 2.4. Data Extraction

Three independent reviewers (AAL, MMS, and RVG) extracted the essential data grouped into five descriptive levels as follows:

- Publication characteristics: author, year, and country
- Characteristics of the animal model: species, sex, age, and weight
- Intervention: control group, dose, frequency, and route and time of administration
- Main results after treatment
- Secondary outcomes

Any disagreements over the extracted data were resolved during discussions with three additional reviewers (SLPM, FCSAM, and RDN).

### 2.5. Bias Analysis

The quality of the studies was assessed through the risk of bias (RoB) tool of SYRCLE (Systematic Review Centre for Laboratory Animal Experimentation) [24]. The following methodological domains based on RoB were evaluated: (1) selection bias (random sequence generation, baseline characteristics, allocation concealment), (2) performance bias (random housing and blinding of caregivers and/or investigators), (3) detection bias (random outcome assessment, blinding of outcome assessment), (4) attrition bias (incomplete outcome data), (5) reporting bias (selective outcome reporting), and other bias (the ethics, statistics, and workplace safety committee). We constructed a figure in Review Manager 5.3 program, from Cochrane Collaboration (RoB 2.0), to demonstrate the risk of bias across all included studies. The items in the RoB tool were scored with “yes” (low risk of bias); “no” (high risk of bias); or “unclear” (indicating that the item was not reported and, therefore, the risk of bias was unknown).

## 3. Results

### 3.1. Selection of PRISMA-Guided Studies

Our search strategy allowed the recovery of 3,067 studies (MEDLINE: 614; Scopus: 1,069; Web of Science: 1,384). After removing 676 duplicates, 2,327 studies were excluded due to inappropriate topic screened by reading the titles and abstracts. Sixty-four studies were completely read (full text), and 44 were excluded through the eligibility criteria. The bibliographical references of the 20 selected articles were analyzed, and 2 studies were added according to the inclusion criteria, resulting in 22 studies [25–46] included in this systematic review (Figure 1).

### 3.2. Characteristics of Publications and Experimental Animals

The general characteristics of the selected studies and experimental models are shown in Table S2. The studies were published between 1955 and 2016 and were conducted in several countries, mainly United States of America (*n* = 8; 36.36%), followed by Germany (*n* = 2; 9.09%), and England, Australia, Korea, Canada, Japan, New Zealand, China, Poland, Nigeria, Iran, Malaysia, and India (*n* = 1 each; 4.54%). Rats were the main animal model used in the studies (*n* = 15; 68.18%), followed by mice (*n* = 7; 31.81%). Among rat strains, most studies used Sprague-Dawley (*n* = 7; 31.81%), followed by Long-Evans (*n* = 3; 13.63%) and Wistar rats (*n* = 3; 13.63%). Albino (*n* = 1; 4.54%) and Wistar-Unilever rats were used in only 1 study (*n* = 1; 4.54%). Among mice, there was great variation between the strains including Fawn, Wild-type, ArKO, apolipoprotein *E*-null, ICR, CD-1, and Balb/C (*n* = 1 each; 4.54%), and only one study (*n* = 1; 4,54%) did not identify the strain.

All experimental animals analyzed were male with variations in the weight and age. All studies with rats reported the animals' weight and age, which varied from 62 to 570 g and from 21 to 365 days, respectively. In mice, the animals' weight ranged from 25 to 43 g, and the age varied from 21 to 42 days; only one study did not report the age, and another did not report the weight.

### 3.3. Characteristics of the Experimental Design

Most studies (*n* = 13; 59.09%) used isoflavone-free feed, eight studies (36.36%) used standard diet, and one study (4.54%) used diet 86 (Table S2). The origin of the chow was mentioned by nineteen studies (86.36%), and three studies (13.63%) did not provide this information (Table S2).

The description of the main experimental characteristics of the studies is detailed in Table S3. Studies have shown variations regarding the beginning of experimental exposure to isoflavones, where ten studies (45.45%) started the exposure during embryonic development (gestation), two studies (9.09%) started in the juvenile phase (28 to 30 days), nine studies (40.90%) started in the adult phase (42 to 180 days), and only one (4.54%) did not mention the animals' initial age. Oral administration of isoflavones was used by most of the studies 90.09% (*n* = 20), of which 68.18% (*n* = 15) used food intake and 22.72% (*n* = 5) gavage. Subcutaneous and intraperitoneal routes were used only in 4.54% of the studies (*n* = 1, each). All studies had a control group in their experiments. The isoflavone-free feed was used as control in nine studies (40.90%); casein in three (13.63%); distilled water and olive oil in two (9.09%, each); and diet 86, standard diet, commercial food, corn oil, Tween-80, and dimethylsulfoxide in one study (4.54%, each). Most of the isoflavones used were purchased from specialized industries (*n* = 16; 72.72%), three were extracted from plants (16.63%), one study used synthetic isoflavone (4.54%) and two studies (9.09%) did not provide this information. The administration of isoflavones was performed on a daily basis in all experiments. The experimental period was reported to be between 5 and 365 days of exposure in rats (*n* = 15; 68.18%) and between 25 and 180 days in mice (*n* = 7; 31.81%).

### 3.4. Isoflavones Characteristics

**Figure 2:**
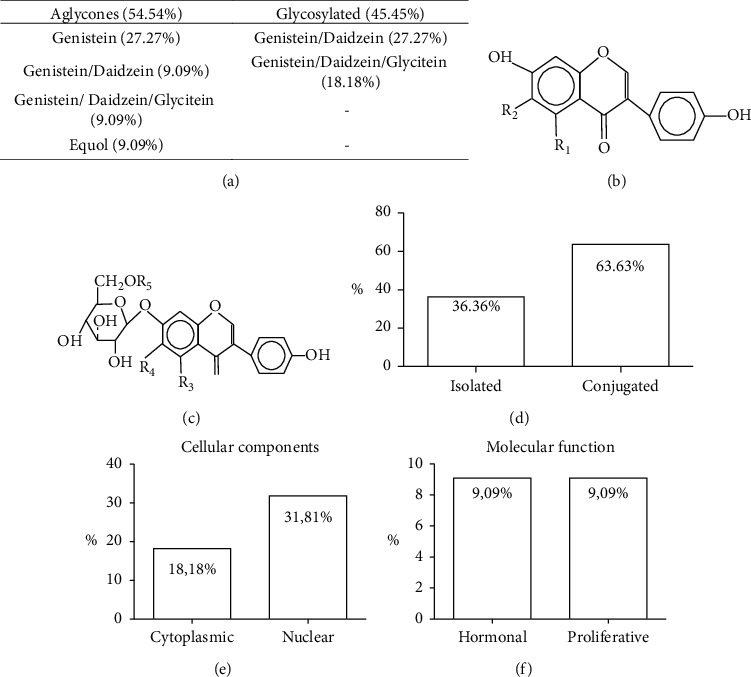
Main forms of isoflavones administration and their physiological changes in murine models: (a) percentage of isoflavones used; (b) chemical form of the aglycone isoflavone; (c) chemical form of the glycosylated isoflavone; (d) percentage of the isoflavones administered, isolated and conjugated; (e, f) main biological processes that led to physiological changes.

The percentage of the main forms of isoflavones administration and their physiological changes in murine models are shown in Figure 2. Four types of isoflavones (genistein, daidzein, glycitein, and equol) were used in the selected studies. Isoflavones were administered separately and conjugated. Aglycone isoflavones were used in over half of the studies (54.54%) and glycosylated in 45.45% of the studies (Figures 2(a)–2(c)). The isolated isoflavone aglycones administered were genistein (*n* = 6; 27.27%) and equol (*n* = 2; 9.09%), and the conjugated aglycones administered were genistein/daidzein (*n* = 3; 13.64%) and genistein/daidzein/glycitein (*n* = 2; 9.09%). All glycosylated isoflavones were administered conjugately, with genistein/daidzein (*n* = 6; 27.27%) and genistein/daidzein/glycitein (*n* = 3; 18.18%) being the most used. The conjugated isoflavones were administered by 63.63% (*n* = 14) of the studies and the isolated by 36.36% (*n* = 10) of the studies (Figure 2(d)). The biological processes of isoflavones action in cellular components occurred in eleven studies (50%) (Figure 2(e)), wherein four studies (18.18%) reported cytoplasmic alterations and seven studies (31.81%) nuclear alterations. Regarding molecular function, the biological processes of isoflavones action were reported by four studies (18.18%) (Figure 2(f)), and in 9.09% (*n* = 2; each) of the studies, the effects of isoflavones on Leydig cells proliferation and hormonal regulation were observed.

### 3.5. Main Results

**Figure 3:**
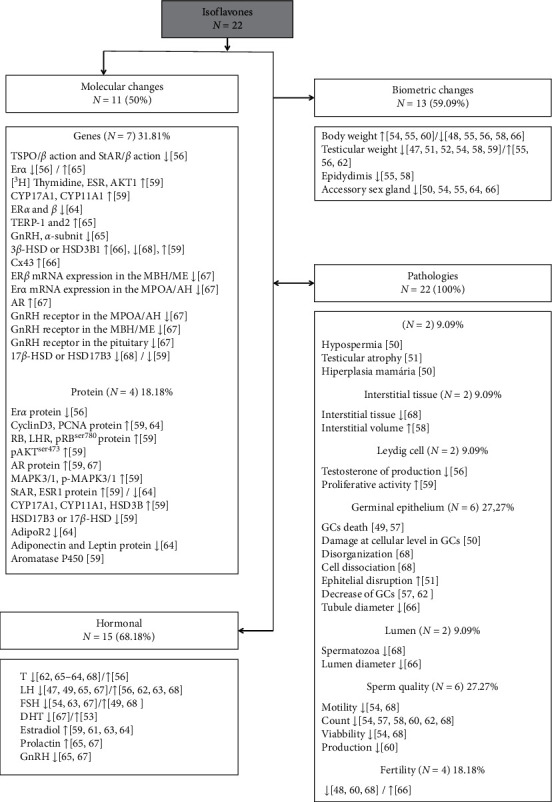
General results of included studies showing the action of isoflavones on testicular function in murine models. GCs: germ cells; ↑: increased; ↓: decreased.

**Figure 4:**
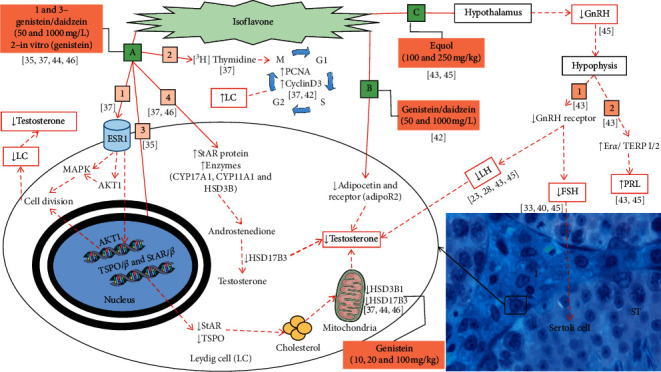
Main biological processes that induce testicular changes after exposure to isoflavones in murine models. Molecular functions involving the nucleus of the Leydig cells and cytoplasmic components (a, b). Molecular functions involving changes in the hypothalamic-pituitary gonadal axis (HPG) (c). I: intertubular; ST: seminiferous tubule.

General results and percentage of molecular, hormonal, biometric, and pathological changes found by the included studies showing the effects of isoflavone on testicular function in murine models are shown in Figure 3. Molecular changes were observed in 50% (*n* = 11) of the studies whose interference in gene activity was found in seven studies (31.81%) and changes in protein synthesis and activity were reported in four studies (18.18%). Hormonal changes were observed in 68.18% of the studies (*n* = 15) and biometric changes in 59.09% of the studies (*n* = 13). Pathological changes were found in all studies selected in this review. The changes in biological processes found in the testicular structure after exposure to isoflavones have been described in detail in Figure 4, showing greater evidence of changes in molecular functions and cellular components.

#### 3.5.1. Biological Processes

The exposure of male rats to isoflavones increased the levels of estrogen receptor *α* (ESR1), mitogen-activated protein kinase 3/1 (MAPK 3/1), protein kinase B (AKT1), pAKTSer473, and [3H] Thymidine in LC (*n* = 1; 4.54%). The same study also observed an increase in Cyclin D3, nuclear cell antigen proliferation (PCNA) proteins (*n* = 2, 9.09%), and decreased expression of translocator protein/*β* (TSPO/*β*) and steroidogenic acute regulatory protein/*β* (StAR/*β*) *in vitro* (Figure 3). Testicular changes due to the action of isoflavones by molecular functions, involving the biological process of regulation of the intracellular estrogen receptor signaling pathway (GO: 0033146) and cell proliferation (GO: 0042127), were found in three (13.63%) studies and are shown in Figure 4(a). Isoflavones (genistein/daidzein/glycitein) can adhere to the estrogen receptor *α* (ESR1) and act through estrogen response elements in the Leydig cells (LC) nucleus to regulate and decrease gene expression and/or induce protein-protein interactions in the signaling pathways. The proteins in the signaling pathways are mediated by protein kinase B (AKT1) and mitogen-activated protein kinase (MAPK) that influence kinase activation and act on the progression of the cell cycle. The modulation of proliferative activity causes a decrease in the LC and, consequently, in the levels of intratesticular and serum testosterone (Figure 4(a), -1). Curiously, *in vitro* exposure to genistein, in the perinatal period, increases the uptake of [3H] Thymidine leading to an increase in the proportion of nuclear cell antigen proliferation (PCNA) and protein cyclin D3 with a consequent increase in LC proliferation (Figure 4, 2). The in vitro study was carried out on studies that performed in vivo analysis, as a way of confirming the results. In addition, the same studies observed an increase in the expression of proteins for the LH receptor (LHR) and ESR1 in the LC and androgen receptor (AR) (5 mg/L) (Figure 3). These results confirm the mitogenic capacity of genistein in LC.

Alteration in proteins involved in cholesterol transport to the mitochondria and, consequently, testosterone synthesis was reported by four (18.18%) studies (Figure 3). The isoflavone genistein/daidzein/glycitein (50 and 1000 mg/L), administered to pregnant rats only during the gestational day (GD) 12 to the postnatal day (PND) 21, induced a reduction in the expression of the steroidogenic acute regulatory protein (StAR) and translocator protein (TSPO). The proteins transcribed by StAR and TSPO, which suffered a decrease, act directly in the transport of cholesterol to the mitochondria. The decrease in cholesterol-carrying proteins in the mitochondria induces a reduction in testosterone production (Figure 4(a), 3) and changes in the phenotype that makes spermatogenesis possible (MP: 0001155).

The expression of 3-beta-hydroxysteroid dehydrogenase (HSD3B1) and 17-beta-hydroxysteroid dehydrogenase (HSD17B3) is involved in the androgen biosynthetic process (GO:0006702) (Figures 4(a), 3 and 4). The expression of HSD3B1, which produces androstenedione in the mitochondria, was more intense in the testes treated with genistein (10 and 100 mg/kg) in the juvenile phase (*n* = 1; 4.54%) than in rats treated during adulthood (*n* = 1; 4.54%) (Figure 4(a), 3). On the other hand, the levels of HSD17B3, which express enzymes that convert androstenedione to testosterone in the mitochondria, decreased when exposed to genistein/daidzein/glycitein (50 and 1000 mg/L) (*n* = 1; 4.54%) and genistein (20 and 100 mg/kg) (*n* = 1; 4.54%) (Figure 4(a), 3). The main consequences of this process are changes in the balance of steroidogenic synthesis. Expression of StAR and other proteins with enzymatic functions such as cytochrome P45017*α*-hydroxylase/17–20 lyase (CYP17A1) and cytochrome P450 (CYP11A1), which are involved in the biological process: steroid biosynthetic process (GO:0006694), had changes in four (18.18%) studies after exposure to isoflavones (Figure 3). Expression of StAR and other proteins with enzymatic functions (CYP17A1, CYP11A1, and HSD3B1) increased after exposure to genistein/daidzein (50 and 1000 mg/L) (*n* = 1; 4.54%) (Fig. 4(a), 4). Isoflavones induce an increase in proteins and enzymes that play an important role in androstenedione production and a decrease in enzyme HSD17B3 that acts as a cofactor in the conversion of androstenedione to testosterone, leading to a decrease in this hormone (Figure 4(a), 4). These changes lead to decreased testosterone production by LC.

The decrease in gene expression of estrogen alpha receptors (Er*α*) was reported in one study (4.54%), where animals were exposed to genistein and daidzein (5 and 1000 mg/L), while an increase was reported in animals exposed to equol (250 mg/kg) indicating endogenous competition (Figure 3). We observed increased expression of the connexin 43 gene (Cx43) in the Leydig cells of animals treated with genistein (10 and 100 mg/kg) in one study (4.54%) (Figure 3). The protein expressed by the Cx43 gene acts on cell-cell communication, allowing it to receive signals from molecules important for its normal development.

The route by which isoflavones promote changes that lead to an endocrine imbalance in the body is shown in Figure 4(b). Isoflavones can induce a reduction in the adiponectin protein and AdipoR2 receptor and, consequently, reduce testosterone production. The adiponectin protein and AdipoR2 gene have the molecular function of protein-hormone receptor activity (GO:0016500). The exposure of male rats to genistein/daidzein (1000 mg/L) in the perinatal period induced a decrease in the adiponectin and AdipoR2 protein (*n* = 1; 4.54%), affecting the endocrine function of Leydig cells (Figure 3). In the same study, the animals were evaluated until adulthood, and testosterone reduction was observed, demonstrating the importance of the adiponectin and AdipoR2 proteins.

Testicular changes due to the action of isoflavones on the molecular functions in the hypothalamic-pituitary-gonadal axis (HPG), that regulates the production of hormones important for spermatogenesis, have been reported by two (9.09%) studies and are shown in Figure 4(c). Gonadotropin-releasing hormone (GnRH) has the molecular function of hormone activity (GO:0005179). Animals treated with equol (250 mg/Kg) showed decreased GnRH production in the hypothalamus and decreased GnRH receptors in the pituitary, leading to decreased LH production and consequently testosterone (Figure 4(c), 1). The FSH also decreased (*n* = 3; 13.63%) (Figure 3), leading to decreased Sertoli cell function. After exposure to equol (100 and 250 mg/kg), there was also a reduction in GnRH which led to an increase in the expression of the Er*α* and truncated estrogen receptor product-1/-2 (TERP-1/2) genes (Figure 3), inducing an increase in prolactin production (Figure 4(c), 2).

Equol at a dose of 250 mg/kg also caused a reduction in messenger ribonucleic acid (mRNA) expression of genes responsible for inducing the production of androgens important for testicular function in specific areas of the brain (*n* = 1, 4.54%) (Figure 3). Doses of 100 and 250 mg/kg reduced the levels of GnRH mRNA in the medial preoptic area/anterior hypothalamus (MPOA/AH), and the dose of 100 mg/kg reduced the levels of expression of ER*α* mRNA (Figure 3). The equol dose of 250 mg/kg reduced the levels of ER*β* mRNA transcripts in the medial basal hypothalamus/median eminence (MBH/ME) (Figure 3). These changes lead to altered mating behavior and decreased GnRH which is important for LH and FSH production.

The action of isoflavones as endocrine disruptors has been observed in most studies (*n* = 15; 68.18%) (Figure 3). Testosterone decreased in nine studies after exposure to all types of tested isoflavones (40.90%). The increase in testosterone levels was reported only in one study (4.54%) where the animals were treated with glycosylated genistein/daidzein/glycitein. The LH, important for the functioning of Leydig cells, decreased in four studies (18.18%) with aglycone (equol and genistein) and glycosylated (genistein/daidzein/glycitein) isoflavones. LH also increased in four studies (18.18%) with aglycone (genistein) and glycosylated (genistein/daidzein/glycitein and genistein/daidzein) isoflavones. The FSH, important for the functioning of Sertoli cells, decreased in three studies (13.63%) with aglycone (equol) and glycosylated (genistein/daidzein/glycitein and genistein/daidzein) isoflavones. The increase in FSH was reported by two studies (9.09%) with aglycone (genistein) and glycosylated (genistein/daidzein) isoflavones. Dihydrotestosterone (DHT), a secondary metabolite of testosterone, decreased in only one study (4.54%) with equol and increased in another study (4.54%) with aglycone (genistein/daidzein/glycitein) isoflavone. Estradiol increased in four studies (18.18%) with aglycone (genistein/daidzein) and glycosylated (genistein/daidzein/glycitein and genistein/daidzein) isoflavones. Prolactin increased in two (9.09%) studies, and GnRH, an important hormone to produce LH and FSH, decreased in two (9.09%) studies, with all animals being treated with equol

Regarding body biometrics, only eight studies (36.36%) reported these values after treatment. Five studies (22.73%) found a significant decrease in body weight of treated animals compared to the control group, and three studies (13.64%) found a significant increase in this parameter. Testicular weight was reported in nine studies (40.90%), decreasing in six (27.27%) and increasing in three (13.63%) of them. The weight of the accessory glands was also reported, with reduction after treatment in five studies (22.73%), and the epididymis weight reduced in two studies (9.09%).

All the selected studies in this review found pathologies after exposure to isoflavones (Figure 3). The main consequences for testicular tissue may have occurred due to changes in molecular function and cellular components. The changes found in the germinal epithelium (*n* = 6; 27.27%) were death, decrease, and damage at the level of germ cells, in addition to disorganization, cell dissociation, and increased epithelial disturbance. Two studies (9.09%) found changes in the interstitial tissue such as decreased tissue (*n* = 1) and increased volume (*n* = 1). A decrease in the number of sperm in the lumen (*n* = 1; 4.54%) and an increase in the luminal diameter (*n* = 1; 4.54%) were also found. Changes in sperm quality (*n* = 6; 27.27%) such as decreased count (*n* = 6), motility (*n* = 2), and viability (*n* = 2) were also observed. In 9.09% (*n* = 2) of the studies, a decrease in testosterone production by Leydig cells was reported. Two studies (9.09%) observed pathologies such as hypospermia, testicular atrophy, and breast hyperplasia. Fertility analysis was performed in four studies (18.18%), decreasing in three studies (13.63%) and increasing in only one (4.54%).

#### 3.5.2. Relationship between Isoflavone Classes and Doses

**Figure 5:**
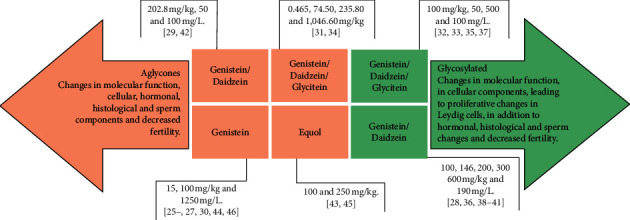
Representation of different doses of isoflavones and their implications for testicular changes.

Figure 5 shows the representation of different doses of isoflavones and their changes in testicular functions. The main changes found in the testicular structure after exposure to aglycone were in molecular functions and cellular components which led to histological damage, sperm changes, and decreased fertility. The animals that received genistein at doses of 15 and 100 mg/kg and 1250 mg/L showed changes in cellular, pathological, and histological components; sperm changes; and decreased fertility. The animals treated with equol at doses of 100 and 250 mg/kg showed changes in cell and hormonal components. The conjugated aglycone genistein/daidzein at doses of 202.8 mg/kg and 50 and 100 mg/L induced changes in cellular components and pathologies. Genistein/daidzein/glycitein at doses of 0.465, 74.50, 235.80, and 1,046.60 mg/Kg showed hormonal, histological, and sperm changes.

The main changes induced by glycosylated isoflavones were in molecular functions; cellular, hormonal, and histological components; sperm changes; and decreased fertility. The animals that received genistein/daidzein at doses of 100, 146, 200, 300, and 600 mg/kg and 190 mg/L showed hormonal and histological changes and decreased fertility. The isoflavone genistein/daidzein/glycitein at doses of 100 mg/kg, 50, 500, and 100 mg/L induced changes in molecular functions and cellular and hormonal components, in addition to histological and sperm changes. Large variations were found in the tested dosages and all showed testicular changes. The dosages of the aglycone isoflavones ranged from 0.465 to 1,046.60 mg/kg and 190 to 1250 mg/L. Glycosylated dosages ranged from 100 to 600 mg/Kg and 100 to 1000 mg/L.

### 3.6. Risk of Bias

**Figure 6:**
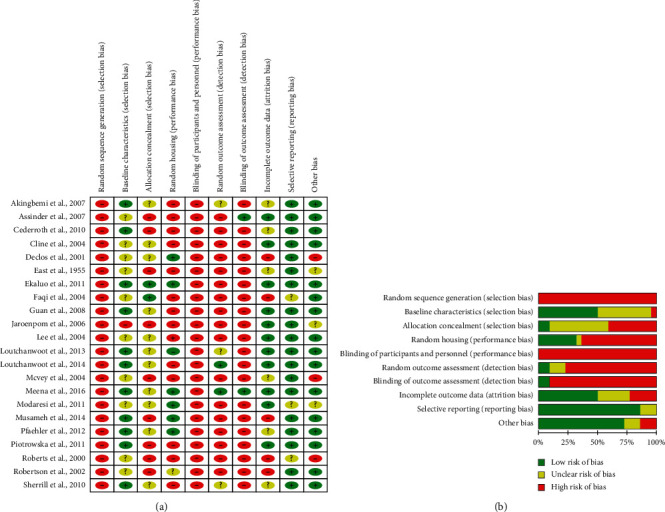
Results of the risk of bias and methodological quality indicators at an individual level (a) and all included studies in this systematic review (b) that evaluated the effect of isoflavone in testicular structure. The items in the Systematic Review Centre for Laboratory Animal Experimentation (SYRCLE) risk of bias assessment were scored with “yes” indicating low risk of bias, “no” indicating high risk of bias, or “unclear” indicating that the item was not reported, resulting in an unknown risk of bias.

Quality assessment at an individual level is shown in Figure 6(a), and the risk of bias assessment of the studies included in this systematic review is shown in Figure 6(b). None of the studies fulfilled all the methodological criteria analyzed. Twenty-one studies (95.45%) described the baseline characteristics (strain, weight, and age) among animals; however, eight studies (*n* = 8; 36.36%) did not present all the baseline characteristics. Only one study (4.54%) did not mention whether the experimental groups were similar in the baseline characteristics. Nine studies (40.90%) did not mention allocation concealment. Three studies (13.64%) reported random animal housing and described the used methods. Five studies (22.73%) only reported that they were randomized, and fourteen studies (63.63%) did not provide this information. Only eight studies (36.36%) reported that animals were exposed to standard environmental conditions, and fourteen studies (64.63%) did not provide this information. None of the studies reported blinding of participants and personnel. Random selection for outcome assessment was applied on six studies (27.27%), and two studies (9.09%) reported blinding outcome assessment. Regarding incomplete outcome data, seven studies (31.81%) did not report if animals were excluded or the reason for the exclusion, seven studies (31.81%) did not clearly report this information, and eight studies (36.36%) mentioned all necessary exclusion information. All studies followed the methodology and presented the proposed results. Most studies (*n* = 20; 90.09%) were apparently free from other problems that could result in a high risk of bias. On the other hand, two studies (9.09%) were at risk due to the omission of the ethics committee of animal studies and the lack of information regarding the statistics used.

## 4. Discussion

In this study, we conducted a systematic review to investigate the action of isoflavones in the testicles of murine models. Our results showed that aglycones and glycosylated isoflavones are harmful to the testicles, whereas glycosylated doses of 50, 100, 146, 200, 300, 500, and 600 mg/kg, as well as 190 and 1000 mg/L, were more harmful. Several biological processes can be altered after exposure to isoflavones, and these processes have been reported in the literature. The main target of isoflavones is the Leydig cells, where decreased testosterone production has been reported more frequently. In addition, the most used route of isoflavones administration was the oral one, showing a concern for simulating the most common form of human exposure.

Regarding the chemical classification of isoflavones, aglycones were mostly used. However, the administration of aglycone and glycosylated conjugates was used by most studies. Aglycones are easily absorbed by the body, whereas glycosylated conjugates undergo previous hydrolysis where they are converted to aglycones by *β*-glycosidases from midgut before the absorption [47, 48]. The administration of conjugated isoflavones may also compromise findings of individual studies, since it is impossible to attribute its effect to a particular type of isoflavone that has been administered in the conjugated form. On the other hand, the isolated compounds provide less generic results that allow better understanding of the mechanisms of action of a specific isoflavone. Among the evaluated studies, genistein and daidzein were the most used, being administered separately and in combination because of the predominant presence of these compounds in soy, a leguminous plant present in large proportions in the human diet [2].

Our findings make it clear that there is concern about isoflavone consumption and reproductive health since 1955 because isoflavones can act as xenoestrogens [49, 50]. Some studies have indicated that the consumption of isoflavones can cause androgenic changes, and others have reported that these changes may be insufficient to impair the testicular function. However, it is known that changes in testicular androgenic control directly impair spermatogenesis [51, 52]. There are reports in the literature indicating that isoflavones inhibit androgen biosynthesis when taken in higher doses [53] or cause an increase in serum testosterone levels when taken in lower concentrations [54, 55]. The biggest changes found in the included studies were in animals that received isoflavones at doses of 50, 100, 146, 200, 300, 500, and 600 mg/kg and 190 and 1000 mg/L, with an exposure period varying from pregnancy to adulthood. We observed that the intake of isoflavones in varying doses can negatively interact with testicular androgenic balance.

Testosterone production decreased significantly due to the action of isoflavones on the HPG axis. Leydig cells (LC) are abundant in the testes with the function of producing testosterone, an essential hormone for the functioning of spermatogenesis and expression of male secondary sexual characteristics [11]. Isoflavones (genistein/daidzein/glycitein) can adhere to ESR1 and regulate the hormonal action in the LC nucleus. The proteins in the signaling pathways are mediated by protein kinase B (AKT1) and mitogen-activated protein kinase (MAPK) that influence kinase activation and act on the progression of the cell cycle. Isoflavones can regulate gene expression and/or induce protein-protein interactions, increasing the phosphorylation of MAPK in MAPK3/1 (p-MAPK3/1 or p-p42/44) and decreasing cell cycle progression. The modulation of hormonal action and proliferative activity cause decreased number and function of LC with a consequent reduction in testicular and serum testosterone levels. Spermatogenesis requires high concentrations of testosterone [11, 52], and low levels of this hormone may indicate reduced testicular function. Together, the studies reported that the *in vitro* exposure to genistein, in the perinatal period, increases the uptake of [3H] Thymidine leading to an increase in the proportion of nuclear cell antigen proliferation (PCNA) and protein cyclin D3 with a consequent increase in LC proliferation. Proteins PCNA and cyclin D3 are important in activating the cell cycle [56, 57]. In addition, these studies observed an increase in the expression of proteins for the LH receptor (LHR) and ESR1 in the LC and androgen receptor (AR) (5 mg/L). These results confirm the mitogenic capacity of genistein in LC and the fact that the exposure of male rats to isoflavones can affect the LC differentiation inducing potential changes in the testicular function.

The reduction in testosterone may also be justified by the decreased expression of mitochondrial TSPO and StAR after exposure to glycosylated isoflavones (genistein/daidzein/glycitein). TSPO and StAR genes are directly associated with the transport and movement of cholesterol across mitochondrial membranes of LC [58]. The decrease in proteins expressed by these genes reduces the transport of cholesterol to the mitochondria of LC and leads to a decrease in the production of testosterone. However, inhibition of the StAR gene was also observed in the presence of large amounts of adiponectin which acts directly on the LH, inhibiting transcription and decreasing androgen secretion. LH stimulates the functioning of Leydig cells [52, 59], and its reduction can lead to a decrease in testosterone production.

Maintaining balance of enzymes that participate in androgen synthesis is important for the normal functioning of the testicles [60]. Leydig cells have a large amount of smooth endoplasmic reticulum and mitochondria which contain enzymes associated with steroid synthesis [60]. Our findings revealed that the aglycone genistein decreased the levels of the enzymes HSD3B1 and HSD17B3. Glycosylated isoflavones (genistein/daidzein/glycitein) increased levels of CYP11A1, HSD3B, and CYP17A1. When cholesterol reaches the mitochondrial matrix, it is cleaved by the enzyme CYP11A1 in pregnenolone, which diffuses from the mitochondria and can be used for the formation of steroid hormones such as estrogen [61]. We also observed that glycosylated isoflavones (genistein/daidzein) induce an increase in serum estradiol levels. The HSD3B1 enzyme converts pregnenolone to progesterone [62, 63]; both are precursors of testosterone biosynthesis and later estradiol [61]. For androgen synthesis, a cascade of enzymes is required to induce the conversion of progesterone to 17*α*-hydroxyprogesterone and then to androstenedione [61–63]. Finally, androstenedione is converted through the action of the HSD17B3 enzyme into testosterone [63, 64]. These changes lead to decreased testosterone production by LC. The changes indicate that isoflavones can increase estradiol levels and decrease testosterone levels, causing an imbalance of cellular functions in males.

The increase in estradiol can also be justified by the increase in aromatase P450 which catalyzes the biosynthesis of estradiol using testosterone as substrate [65]. Therefore, high levels of estradiol due to ingestion of isoflavones may indicate greater availability of substrate as a result of increased activity of aromatase P450. The adipose tissue, which is the main source of serum estrogen in males, in addition to expressing aromatase P450 has estrogen receptors (ERs) that are targets for receiving antagonistic stimuli [65]. Thus, isoflavones can bind to ERs in adipose tissue to regulate estrogen biosynthesis [65].

The protein expressed by the Cx43 acts on cell-to-cell communication allowing the signaling of important molecules for cellular development [66, 67]. In the included studies, the expression of Cx43 gene was higher in LC, seminiferous epithelium, spermatogonia, spermatocytes, and spermatozoa of animals treated with isoflavone aglycone (genistein). The protein expressed by Cx43 gene is strongly associated with Sertoli cells maturation, proliferation, and development of germ cells [68, 69]. The increased expression of Cx43 indicates that genistein did not damage the development of the gonads in these animals.

The present findings revealed that the hormonal balance was altered by all the isoflavones used. However, changes in molecular functions via HPG axis were reported only for the aglycone isoflavone. Equol exposure reduced gonadotropin-releasing hormone receptor (GnRHR) gene expression, ER*α* mRNA, TERP-1 and TERP-2, luteinizing hormone beta subunit (LH*β*) gene, and gonadotropin alpha subunit. The expression of TERP-1/-2 in rats is sensitive and positively regulated by estradiol [70, 71], indicating isoflavones' estrogenic action. Equol also induced increased expression of ER*α* mRNA gene, estradiol, and prolactin in the included studies. ER*α* mRNA gene in the pituitary gland is also regulated by estrogen [70, 71]. High levels of estrogen can also inhibit the LC proliferation, induce hyperplasia of the mammary gland in male rats, with an increased risk of developing cancer [72, 73], and decrease testosterone secretion. The increased activity of these genes by the action of isoflavones indicates that equol has estrogenic capacity.

The decrease in LH and FSH levels reported in the included studies can also be justified by the decrease in the GnRHR in the pituitary. The FSH and LH levels keep the testosterone concentration balanced, being essential for the sperm production. [74]. Equol induces a reduction in mRNA expression of the genes responsible for inducing androgen production in specific areas of the brain. We observed a reduction in levels of GnRH mRNA in the MBH/ME and in the MPOA/AH, which has the neurons that promote mating behavior in male rats. In addition, the included studies also reported decrease in the expression of ER*α*, ER*β*, and androgen receptor (AR) genes. The decline in GnRH receptor expression in the pituitary can be explained by an indirect mechanism, via reduced activity of hypothalamic GnRH neurons [70, 71].

Isoflavones can affect testicular structures by acting as endocrine disruptors. Endocrine dysregulation during spermatogenesis leads to the production of defective sperm, with problems of motility, capacity, and viability [75]. The decrease in FSH observed in our findings may cause a reduction in cyclic adenosine monophosphate (cAMP) and androgen-binding protein (ABP) levels compromising spermatogenesis [67]. Studies have also indicated that estrogen intake causes a decrease in LH and FSH serum levels associated with an increase in prolactin serum levels [63]. Hormonal dysfunction can also cause changes in the accessory glands since testosterone acts on the functioning of sexual organs. These results indicate estrogenic activity of all tested isoflavones. As the changes become more severe and apparent, it is possible to observe the action of isoflavones such as decreased testicular and body weight, pathologies, testicular atrophy, hypospermia, and breast hyperplasia.

The analysis of body weight provides important information about the general toxicity of a compound and possible health implications [51]. The animals treated with aglycone and glycosylated isoflavones showed reductions in body, testicular, epididymal, and accessory glands weight. Testicular weight and/or size can be used as indicators of sperm production since its main component is the seminiferous tubule [76]. The changes found indicate testicular toxicity caused by isoflavones which may impair sperm production.

All selected studies found histopathological changes after exposure to isoflavones. We observed that exposure to aglycone and glycosylated isoflavones interrupted spermatogenesis, increased apoptosis in germ cells, and decreased seminiferous epithelium, in addition to causing disorganization and detachment of the seminiferous epithelium. The occurrence of cell death and decreased germ cells is directly reflected in the sperm count and daily sperm production [75, 77, 78]. Other changes such as dissociation, disorganization, decreased cells, and nuclear volume of seminiferous epithelium cells were also found. Changes in the testicular structure can trigger functional changes which lead to infertility [52, 51, 58].

Significant reductions in daily sperm production (DSP) and epididymal sperm count in mice exposed to genistein have been reported. These findings agree with other studies where the exposure of rodents to estrogenic compounds [79, 80] led to the emergence of histopathologies such as hypospermia and testicular atrophy. Animals treated with aglycones (genistein and genistein/daidzein/glycitein) and glycosylated isoflavones showed decreased quality, viability, count, and sperm production. The decrease in sperm quality can result in infertility due to reduced ability of the sperm to reach the fertilization site, as well as its ability to penetrate the pellucid zone [11]. Exposure to isoflavones reduces fertility due to altered expression of steroid receptors and increases sperm lipid peroxidation [75, 80]. However, the evaluated studies did not report the oxidative status of animals exposed to isoflavones.

Systematic reviews are considered high-level studies that allow for the individual evaluation of studies in a blind way using specific tools [81]. Such features lead to a more inclusive and reliable approach, providing a broad understanding of the included studies. However, discrepancies between the studies reviewed and included methods are shown as limitations considering the wide variety in animal models' characteristics such as age, weight, number of animals, and number of experimental groups. Another limitation was the wide variety of doses (0.465 to 2000 mg/kg and 5 to 1250 mg/L). If we take into account the fact that the intake of isoflavones resulting from soy consumption in adult humans is around 1 mg/kg/day and infant formulations around 6 to 9 mg/kg/day [14], the used doses in the included studies were high. However, many foods have traces of soy which increases the level of daily consumption.

## 5. Conclusions

The consumption of aglycones and glycosylated isoflavones was harmful to the testicles, with the glycosylated doses of 50, 100, 146, 190, 200, 300, 500, and 600 mg/kg and 1000 mg/L being more harmful. The administration of isolated isoflavones is more efficient in evaluating their individual effect on testicular structures; however, the conjugated compound was the most used in the studies. The main testicular pathologies resulting from the use of isoflavones are mainly associated with Leydig cells and are the result of changes in molecular functions and cellular components. In addition, high doses of isoflavones showed great changes in testicular structures causing infertility in experimental murine models. Isoflavones have the ability to negatively alter testicular microstructure by acting directly on the expression of genes important for the production of hormones. However, further studies are needed to analyze the oxidative status of the testicles, to understand the action of isoflavones in Leydig cells, and to evaluate the pathway that leads to the alteration of androgen receptors in Sertoli cells and to histopathological changes in the seminiferous epithelium.

## Data Availability

The data supporting the findings of this study are available within the article.

## Supplementary Materials

Supplementary Materials Table S1: search filters used in PubMed, Scopus, and Web of Science databases. Table S2: characteristics of the experimental models and diet used in all studies that evaluated isoflavone on testicular function. Table S3: description of the main experiment.
