# Supplementary material for: Relevance of the Isoflavone Absorption and Testicular Function: A Systematic Review of Preclinical Evidence
Source: Evid Based Complement Alternat Med. 2021 Feb 12;2021:8853172. doi: 10.1155/2021/8853172 (PMC7895610; doi:10.1155/2021/8853172)
Supplement: Supplementary Materials — Table S1: search filters used in PubMed, Scopus, and Web of Science databases. Table S2: characteristics of the experimental models and diet used in all studies that evaluated isoflavone on testicular function. Table S3: description of the main experiment. [file 8853172.f1.zip › 8853172.f1/Supplemental Files - S1.docx]

**Table S1.** Search filters used in PubMed, Scopus and Web of Science databases.

| **DESCRIPTORS OF RESEARCH** | | |
| --- | --- | --- |
| **PUBMED** | | |
| ***LABORATORY ANIMALS PART 1*** | ***TESTIS*** | ***ISOFLAVONE*** |
| ["animal experimentation"[MeSH Terms] OR "models, animal"[MeSH Terms] OR "invertebrates"[MeSH Terms] OR "Animals"[Mesh:noexp] OR "animal population groups"[MeSH Terms] OR "chordata"[MeSH Terms:noexp] OR "chordata, nonvertebrate"[MeSH Terms] OR "vertebrates"[MeSH Terms:noexp] OR "amphibians"[MeSH Terms] OR "birds"[MeSH Terms] OR "fishes"[MeSH Terms] OR "reptiles"[MeSH Terms] OR "mammals"[MeSH Terms:noexp] OR "primates"[MeSH Terms:noexp] OR "artiodactyla"[MeSH Terms] OR "carnivora"[MeSH Terms] OR "cetacea"[MeSH Terms] OR "chiroptera"[MeSH Terms] OR "elephants"[MeSH Terms] OR "hyraxes"[MeSH Terms] OR "insectivora"[MeSH Terms] OR "lagomorpha"[MeSH Terms] OR "marsupialia"[MeSH Terms] OR "monotremata"[MeSH Terms] OR "perissodactyla"[MeSH Terms] OR "rodentia"[MeSH Terms] OR "scandentia"[MeSH Terms] OR "sirenia"[MeSH Terms] OR "xenarthra"[MeSH Terms] OR "haplorhini"[MeSH Terms:noexp] OR "strepsirhini"[MeSH Terms] OR "platyrrhini"[MeSH Terms] OR "tarsii"[MeSH Terms] OR "catarrhini"[MeSH Terms:noexp] OR "cercopithecidae"[MeSH Terms] OR "hylobatidae"[MeSH Terms] OR "hominidae"[MeSH Terms:noexp] OR "gorilla gorilla"[MeSH Terms] OR "pan paniscus"[MeSH Terms] OR "pan troglodytes"[MeSH Terms] OR "pongo pygmaeus"[MeSH Terms]] | [“testis”[MeSH Terms] OR “testicle”[ MeSH Terms] OR “testicles”[MeSH Terms] OR “testes”[ MeSH Terms] OR “spermatogenesis”[MeSH Terms] OR “testosterone”[MeSH Terms] OR “spermatogonia”[MeSH Terms] OR “spermatogonias”[MeSH Terms] OR “spermatocyte”[MeSH Terms] OR “spermatocytes”[MeSH Terms] OR “spermatid”[MeSH Terms] OR “spermatids”[MeSH Terms] OR “leydig cells”[MeSH Terms] OR “seminiferous tubules”[MeSH Terms] OR “blood-testis barrier”[MeSH Terms] OR “seminiferous epithelium”[MeSH Terms] OR “sertoli cells”[MeSH Terms] OR “testis”[TIAB] OR “testicle”[TIAB] OR “testicles”[TIAB] OR “testes”[TIAB] OR “spermatogenesis”[TIAB] OR “testosterone”[TIAB] OR “spermatogonia”[Tiab] OR “spermatogonias”[TIAB] OR “spermatocyte”[TIAB] OR “spermatocytes”[Tiab] OR “spermatid”[Tiab] OR “spermatids”[TIAB] OR “leydig cells”[TIAB] OR “seminiferous tubules”[TIAB] OR “blood-testis barrier”[TIAB] OR “seminiferous epithelium”[TIAB] OR “sertoli cells”[TIAB]] | (TITLE-ABS-KEY("phytoestrogens") OR TITLE-ABS-KEY(“phytoestrogen") OR TITLE-ABS-KEY(“plant estrogen”) OR TITLE-ABS-KEY(“estrogen, plant”) OR TITLE-ABS-KEY(“plant estrogens”) OR TITLE-ABS-KEY(“estrogens, plant”) OR TITLE-ABS-KEY(“phyto-estrogen”) OR TITLE-ABS-KEY(“phyto- estrogen”) OR TITLE-ABS-KEY(“estrogen-like plant extracts”) OR TITLE-ABS-KEY(“estrogen like plant extracts”) OR TITLE-ABS-KEY(“extracts, estrogen-like plant”) OR TITLE-ABS-KEY(“plant extracts, estrogen-like”) OR TITLE-ABS-KEY(“androgen antagonists”) OR TITLE-ABS-KEY(“isoflafone”) OR TITLE-ABS-KEY(“isoflavones”) OR TITLE-ABS-KEY(“isoflavone derivatives”) OR TITLE-ABS-KEY(“derivatives, isoflavone”) OR TITLE-ABS-KEY(“genistein”) OR TITLE-ABS-KEY(“daidzein”) OR TITLE-ABS-KEY(“glycine”) OR TITLE-ABS-KEY(“formononetine”) OR TITLE-ABS-KEY(“biocanin A”) OR TITLE-ABS-KEY(“equol”)) |

**Table S1 (*Continuation*).** Search filters used in PubMed, Scopus and Web of Science databases.

| ***LABORATORY ANIMALS PART 2*** |
| --- |
| [animals[TIAB] OR animal[TIAB] OR mice[TIAB] OR mus[TIAB] OR mouse[TIAB] OR murine[TIAB] OR woodmouse[TIAB] OR rats[TIAB] OR rat[TIAB] OR murinae[TIAB] OR muridae[TIAB] OR cottonrat[TIAB] OR cottonrats[TIAB] OR hamster[TIAB] OR hamsters[TIAB] OR cricetinae[TIAB] OR rodentia[TIAB] OR rodent[TIAB] OR rodents[TIAB] OR pigs[TIAB] OR pig[TIAB] OR swine[TIAB] OR swines[TIAB] OR piglets[TIAB] OR piglet[TIAB] OR boar[TIAB] OR boars[TIAB] OR "sus scrofa"[TIAB] OR ferrets[TIAB] OR ferret[TIAB] OR polecat[TIAB] OR polecats[TIAB] OR "mustela putorius"[TIAB] OR "guinea pigs"[TIAB] OR "guinea pig"[TIAB] OR cavia[TIAB] OR callithrix[TIAB] OR marmoset[TIAB] OR marmosets[TIAB] OR cebuella[TIAB] OR hapale[TIAB] OR octodon[TIAB] OR chinchilla[TIAB] OR chinchillas[TIAB] OR gerbillinae[TIAB] OR gerbil[TIAB] OR gerbils[TIAB] OR jird[TIAB] OR jirds[TIAB] OR merione[TIAB] OR meriones[TIAB] OR rabbits[TIAB] OR rabbit[TIAB] OR hares[TIAB] OR hare[TIAB] OR diptera[TIAB] OR flies[TIAB] OR fly[TIAB] OR dipteral[TIAB] OR drosphila[TIAB] OR drosophilidae[TIAB] OR cats[TIAB] OR cat[TIAB] OR carus[TIAB] OR felis[TIAB] OR nematoda[TIAB] OR nematode[TIAB] OR nematoda[TIAB] OR nematode[TIAB] OR nematodes[TIAB] OR sipunculida[TIAB] OR dogs[TIAB] OR dog[TIAB] OR canine[TIAB] OR canines[TIAB] OR canis[TIAB] OR sheep[TIAB] OR sheeps[TIAB] OR mouflon[TIAB] OR mouflons[TIAB] OR ovis[TIAB] OR goats[TIAB] OR goat[TIAB] OR capra[TIAB] OR capras[TIAB] OR rupicapra[TIAB] OR chamois[TIAB] OR haplorhini[TIAB] OR monkey[TIAB] OR monkeys[TIAB] OR anthropoidea[TIAB] OR anthropoids[TIAB] OR saguinus[TIAB] OR tamarin[TIAB] OR tamarins[TIAB] OR leontopithecus[TIAB] OR hominidae[TIAB] OR ape[TIAB] OR apes[TIAB] OR pan[TIAB] OR paniscus[TIAB] OR "pan paniscus"[TIAB] OR bonobo[TIAB] OR bonobos[TIAB] OR troglodytes[TIAB] OR "pan troglodytes"[TIAB] OR gibbon[TIAB] OR gibbons[TIAB] OR siamang[TIAB] OR siamangs[TIAB] OR nomascus[TIAB] OR symphalangus[TIAB] OR chimpanzee[TIAB] OR chimpanzees[TIAB] OR prosimians[TIAB] OR "bush baby"[TIAB] OR prosimian[TIAB] OR bush babies[TIAB] OR galagos[TIAB] OR galago[TIAB] OR pongidae[TIAB] OR gorilla[TIAB] OR gorillas[TIAB] OR pongo[TIAB] OR pygmaeus[TIAB] OR "pongo pygmaeus"[TIAB] OR orangutans[TIAB] OR pygmaeus[TIAB] OR lemur[TIAB] OR lemurs[TIAB] OR lemuridae[TIAB] OR horse[TIAB] OR horses[TIAB] OR pongo[TIAB] OR equus[TIAB] OR cow[TIAB] OR calf[TIAB] OR bull[TIAB] OR chicken[TIAB] OR chickens[TIAB] OR gallus[TIAB] OR quail[TIAB] OR bird[TIAB] OR birds[TIAB] OR quails[TIAB] OR poultry[TIAB] OR poultries[TIAB] OR fowl[TIAB] OR fowls[TIAB] OR reptile[TIAB] OR reptilia[TIAB] OR reptiles[TIAB] OR snakes[TIAB] OR snake[TIAB] OR lizard[TIAB] OR lizards[TIAB] OR alligator[TIAB] OR alligators[TIAB] OR crocodile[TIAB] OR crocodiles[TIAB] OR turtle[TIAB] OR turtles[TIAB] OR amphibian[TIAB] OR amphibians[TIAB] OR amphibia[TIAB] OR frog[TIAB] OR frogs[TIAB] OR bombina[TIAB] OR salientia[TIAB] OR toad[TIAB] OR toads[TIAB] OR "epidalea calamita"[TIAB] OR salamander[TIAB] OR salamanders[TIAB] OR eel[TIAB] OR eels[TIAB] OR fish[TIAB] OR fishes[TIAB] OR pisces[TIAB] OR catfish[TIAB] OR catfishes[TIAB] OR siluriformes[TIAB] OR arius[TIAB] OR heteropneustes[TIAB] OR sheatfish[TIAB] OR perch[TIAB] OR perches[TIAB] OR percidae[TIAB] OR perca[TIAB] OR trout[TIAB] OR trouts[TIAB] OR char[TIAB] OR chars[TIAB] OR salvelinus[TIAB] OR "fathead minnow"[TIAB] OR minnow[TIAB] OR cyprinidae[TIAB] OR carps[TIAB] OR carp[TIAB] OR zebrafish[TIAB] OR zebrafishes[TIAB] OR goldfish[TIAB] OR goldfishes[TIAB] OR guppy[TIAB] OR guppies[TIAB] OR chub[TIAB] OR chubs[TIAB] OR tinca[TIAB] OR barbels[TIAB] OR barbus[TIAB] OR pimephales[TIAB] OR promelas[TIAB] OR "poecilia reticulata"[TIAB] OR mullet[TIAB] OR mullets[TIAB] OR seahorse[TIAB] OR seahorses[TIAB] OR mugil curema[TIAB] OR atlantic cod[TIAB] OR shark[TIAB] OR sharks[TIAB] OR catshark[TIAB] OR anguilla[TIAB] OR salmonid[TIAB] OR salmonids[TIAB] OR whitefish[TIAB] OR whitefishes[TIAB] OR salmon[TIAB] OR salmons[TIAB] OR sole[TIAB] OR solea[TIAB] OR "sea lamprey"[TIAB] OR lamprey[TIAB] OR lampreys[TIAB] OR |

**Table S1 (*Continuation*).** Search filters used in PubMed, Scopus and Web of Science databases.

| ***LABORATORY ANIMALS PART 2*** | |
| --- | --- |
| pumpkinseed[TIAB] OR sunfish[TIAB] OR sunfishes[TIAB] OR tilapia[TIAB] OR tilapias[TIAB] OR turbot[TIAB] OR turbots[TIAB] OR flatfish[TIAB] OR flatfishes[TIAB] OR sciuridae[TIAB] OR squirrel[TIAB] OR squirrels[TIAB] OR chipmunk[TIAB] OR chipmunks[TIAB] OR suslik[TIAB] OR susliks[TIAB] OR vole[TIAB] OR voles[TIAB] OR lemming[TIAB] OR lemmings[TIAB] OR muskrat[TIAB] OR muskrats[TIAB] OR lemmus[TIAB] OR otter[TIAB] OR otters[TIAB] OR marten[TIAB] OR martens[TIAB] OR martes[TIAB] OR weasel[TIAB] OR badger[TIAB] OR badgers[TIAB] OR ermine[TIAB] OR mink[TIAB] OR minks[TIAB] OR sable[TIAB] OR sables[TIAB] OR gulo[TIAB] OR gulos[TIAB] OR wolverine[TIAB] OR wolverines[TIAB] OR minks[TIAB] OR mustela[TIAB] OR llama[TIAB] OR llamas[TIAB] OR alpaca[TIAB] OR alpacas[TIAB] OR camelid[TIAB] OR camelids[TIAB] OR guanaco[TIAB] OR guanacos[TIAB] OR chiroptera[TIAB] OR chiropteras[TIAB] OR bat[TIAB] OR bats[TIAB] OR fox[TIAB] OR foxes[TIAB] OR iguana[TIAB] OR iguanas[TIAB] OR xenopus laevis[TIAB] OR parakeet[TIAB] OR parakeets[TIAB] OR parrot[TIAB] OR parrots[TIAB] OR donkey[TIAB] OR donkeys[TIAB] OR mule[TIAB] OR mules[TIAB] OR zebra[TIAB] OR zebras[TIAB] OR shrew[TIAB] OR shrews[TIAB] OR bison[TIAB] OR bisons[TIAB] OR buffalo[TIAB] OR buffaloes[TIAB] OR deer[TIAB] OR deers[TIAB] OR bear[TIAB] OR bears[TIAB] OR panda[TIAB] OR pandas[TIAB] OR "wild hog"[TIAB] OR "wild boar"[TIAB] OR fitchew[TIAB] OR fitch[TIAB] OR beaver[TIAB] OR beavers[TIAB] OR jerboa[TIAB] OR jerboas[TIAB] OR capybara[TIAB] OR capybaras[TIAB]) NOT medline[subset]] | |
| **SCOPUS** | |
| ***TESTIS*** | ***ISOFLAVONE*** |
| ((TITLE-ABS-KEY(“testis”) OR TITLE-ABS-KEY(“testicle”) OR TITLE-ABS-KEY (“testicles”) OR TITLE-ABS-KEY(“testes”) OR TITLE-ABS-KEY (“spermatogenesis”) OR TITLE-ABS-KEY(“testosterone”) OR TITLE-ABS-KEY(“spermatogonia”) OR TITLE-ABS-KEY(“spermatogonias”) OR TITLE-ABS-KEY(“spermatocyte”) OR TITLE-ABS-KEY (“spermatocytes”) OR TITLE-ABS-KEY(“spermatid”) OR TITLE-ABS-KEY(“spermatids”) OR TITLE-ABS-KEY(“leydig cells”) OR TITLE-ABS-KEY(“seminiferous tubules”) OR TITLE-ABS-KEY(“blood-testis barrier”) OR TITLE-ABS-KEY(“seminiferous epithelium”) OR TITLE-ABS-KEY(“sertoli cells”)) | (TITLE-ABS-KEY("phytoestrogens") OR TITLE-ABS-KEY(“phytoestrogen") OR TITLE-ABS-KEY(“plant estrogen”) OR TITLE-ABS-KEY(“estrogen, plant”) OR TITLE-ABS-KEY(“plant estrogens”) OR TITLE-ABS-KEY(“estrogens, plant”) OR TITLE-ABS-KEY(“phyto-estrogen”) OR TITLE-ABS-KEY(“phyto- estrogen”) OR TITLE-ABS-KEY(“estrogen-like plant extracts”) OR TITLE-ABS-KEY(“estrogen like plant extracts”) OR TITLE-ABS-KEY(“extracts, estrogen-like plant”) OR TITLE-ABS-KEY(“plant extracts, estrogen-like”) OR TITLE-ABS-KEY(“androgen antagonists”) OR TITLE-ABS-KEY(“isoflafone”) OR TITLE-ABS-KEY(“isoflavones”) OR TITLE-ABS-KEY(“isoflavone derivatives”) OR TITLE-ABS-KEY(“derivatives, isoflavone”) OR TITLE-ABS-KEY(“genistein”) OR TITLE-ABS-KEY(“daidzein”) OR TITLE-ABS-KEY(“glycine”) OR TITLE-ABS-KEY(“formononetine”) OR TITLE-ABS-KEY(“biocanin A”) OR TITLE-ABS-KEY(“equol”)) |

**Table S1 (*Continuation*).** Search filters used in PubMed, Scopus and Web of Science databases

| **WEB OF SCIENCE** | | |
| --- | --- | --- |
| ***ANIMALS*** | ***TESTIS*** | **ISOFLAVONE** |
| TS=Mice OR TS=Mouse OR TS=Rat OR TS=Rats OR TS=Dog OR TS=Dogs OR TS=Rabbits OR TS=Murine model OR TS=Guinea pig OR TS=Hamster OR TS=Animal model | TS=testis OR TS=testicle OR TS=testicles OR TS=testes OR TS=spermatogenesis OR TS=testosterone OR TS=spermatogonia OR TS=spermatogonias OR TS=spermatocyte OR TS=spermatocytes OR TS=spermatid OR TS=spermatids OR TS=leydig cells OR TS=seminiferous tubules OR TS=blood-testis barrier OR TS=seminiferous epithelium OR TS=sertoli cells | TS=phytoestrogens OR TS=phytoestrogen OR TS=plant estrogen OR TS=estrogen, plant OR TS=plant estrogens OR TS=estrogens, plant OR TS=phyto-estrogen OR TS=phyto- estrogen OR TS=estrogen-like plant extracts OR TS=estrogen like plant extracts OR TS=extracts, estrogen-like plant OR TS=plant extracts, estrogen-like OR TS=androgen antagonists OR TS=isoflafone OR TS=isoflavones OR TS=isoflavone derivatives OR TS=derivatives, isoflavone OR TS=genistein OR TS=daidzein OR TS=glycine OR TS=formononetine OR TS=biocanin A OR TS=equol |
