# Supplementary material for: Relevance of the Isoflavone Absorption and Testicular Function: A Systematic Review of Preclinical Evidence
Source: Evid Based Complement Alternat Med. 2021 Feb 12;2021:8853172. doi: 10.1155/2021/8853172 (PMC7895610; doi:10.1155/2021/8853172)
Supplement: Supplementary Materials — Table S1: search filters used in PubMed, Scopus, and Web of Science databases. Table S2: characteristics of the experimental models and diet used in all studies that evaluated isoflavone on testicular function. Table S3: description of the main experiment. [file 8853172.f1.zip › 8853172.f1/Supplemental Files - S2.docx]

**Table S2.** Characteristics of the experimental models and diet used in all studies that evaluated isoflavone on testicular function.

| **Study** | **Country** | **Animal model** | **Strain** | **Sex** | **Weight (g)** | **Age (days)** | **Dietary strategy** | **Diet / Manufacturer** |
| --- | --- | --- | --- | --- | --- | --- | --- | --- |
| East, 1955  [25] | ENG | Mice | Fawn Fawn | Male | (?) | 60 | Isoflavone-free feed | Wholemeal flour 45%, ground oats 40%, fish meal 8%, Dried yeast 1%, dried skimmed milk 3%, Cod-liver oil 2% and sodium chloride 1%, (?) |
| Roberts et al., 2000  [26] | USA | Rats | Sprague-Dawley | Male | 300 | 21 / 70 / 130 | Isoflavone-free feed | (?), American Institute of Nutrition (AIN) |
| Delclos et al., 2001  [27] | USA | Rats | Sprague-Dawley | Male | 63 | 21 / 50 | Isoflavone-free feed | (?), Standard NIH-31 pellet diet |
| Robertson et al., 2002  [28] | AUS | Mice | Wild-type and ArKO | Male | 25 | 98 / 365 | Isoflavone-free feed | (?), Glen Forrest, Western Australia |
| Cline et al., 2004  [29] | USA | Mice | Apolipoprotein E-null | Male | 40 | 42 | Isoflavone-free feed | AIN 76A, (?) |
| Lee et al., 2004  [30] | KOR | Mice | ICR | Male | 35 | 35 | Standard diet | (?), Purina Korea, Seoul, Korea® |
| Mcvey et al., 2004  [31] | CAN | Rats | Sprague-Dawley | Male | 99 | 50 | Isoflavone-free feed | AIN 96G, (?) |
| Faqi et al., 2004  [32] | USA | Rats | Wistar-Unilever | Male | 390 | 42 | Isoflavone-free feed | (?), Teklad 4% Fat Rat/Mouse Diet |
| Jaroenporn et al., 2006  [33] | JPN | Mice | (?) | Male | 42 | 60 | Standard diet | (?), Pokphand, Animal Feed Co., Ltd., Bangkok, Thailand |
| Assinder et al., 2007  [34] | NZL | Rats | Wistar | Male | 570 | 90 | Diet 86 | 78.8% cereal, 1.5% skimmed milk, 7% fish meal, 6% bone meal, 0.5% NaCl, 0.1% rodent premix, and 1% soy meal, Sharpe, Palmerston North, New Zealand |
| Akingbemi et al., 2007  [35] | USA | Rats | Long-Evans | Male | 250 | 21 | Isoflavone-free feed | (?), Tarlan-Heklad, Indianapolis, IN |

**Table S2 (*Continued*).** Characteristics of the experimental models and the diet used in all studies that evaluated isoflavone on testicular function.

| **Study** | **Country** | **Species** | **Lineage** | **Sex** | **Weight (g)** | **Age (days)** | **Dietary strategy** | **Diet / Manufacturer** |
| --- | --- | --- | --- | --- | --- | --- | --- | --- |
| Guan et al., 2008  [36] | CHN | Rats | Sprague-Dawley | Male | 100 | 21 | Standard diet | Australia  Daidzin (0.0011%), genistein (0.0616%), genistin (0.0002%), and daidzein (0.0026%), PicoLab® Rodent Diet 20 |
| Sherrill et al., 2010  [37] | USA | Rats | Long-Evans | Male | 250 | 21 | Standard diet | (?), Tarlan-Heklad, Indianapolis, IN |
| Cederroth et al., 2010  [38] | USA | Mice | CD 1 | Male | 43 | 21 | Isoflavone-free feed | (?), Comercial (Harlan Teklad®) |
| Piotrowska et al., 2011  [39] | POL | Rats | Wistar | Male | 418 | 21 | Standard diet | (?) |
| Ekaluo et al., 2011  [40] | NGR | Rats | Albino | Male | (?) | 90 | Standard diet | (?) |
| Modaresi et al., 2011  [41] | IRI | Mice | Balb/C | Male | 35 | (?) | Isoflavone-free feed | (?) |
| Pfaehler et al., 2012  [42] | USA | Rats | Long-Evans | Male | 62 | 21 | Standard diet | (?), Tarlan-Heklad, Indianapolis, IN |
| Loutchanwoot et al., 2013  [43] | GER | Rats | Sprague-Dawley | Male | 378 | 60 | Isoflavone-free feed | (?), Sniff Spezialdiäten GmbH, Soest, Germany |
| Musameh et al., 2014  [44] | MAS | Rats | Sprague-Dawley | Male | 208 | 21 | Standard diet | (?), Rain Tree, Australia |
| Loutchanwoot et al., 2014  [45] | GER | Rats | Sprague-Dawley | Male | 378 | 60 | Isoflavone-free feed | (?), Ssniff Spezialdiäten GmbH, Soest, Germany |
| Meena et al., 2016  [46] | IND | Rats | Wistar | Male | 281 | 21 | Isoflavone-free feed | (?), Purchased from Sai Durga Agencies, Bengaluru, India |

ENG- England; USA - United States of America; AUS - Australia; KOR - Korea; CAN - Canada; JPN - Japan; NZL – New Zealand; CHN - China; POL - Poland; NGR - Nigeria; IRI - Iran; GER - Germany; MAS - Malaysia; IND - India.
