# Supplementary material for: Relevance of the Isoflavone Absorption and Testicular Function: A Systematic Review of Preclinical Evidence
Source: Evid Based Complement Alternat Med. 2021 Feb 12;2021:8853172. doi: 10.1155/2021/8853172 (PMC7895610; doi:10.1155/2021/8853172)
Supplement: Supplementary Materials — Table S1: search filters used in PubMed, Scopus, and Web of Science databases. Table S2: characteristics of the experimental models and diet used in all studies that evaluated isoflavone on testicular function. Table S3: description of the main experiment. [file 8853172.f1.zip › 8853172.f1/Supplemental Files - S3.docx]

**Table S3.** Description of the main experimental characteristics of the studies that evaluated the action of isoflavones on testicular function in murine models.

| **Study** | **Beginning of treatment** | **Route** | **Control** | **Treated/Isoflavone-enriched diet** | **Frequency** | **Duration**  **(Days)** |
| --- | --- | --- | --- | --- | --- | --- |
| East, 1955  [25] | 60 days | Oral  (Food intake) | Isoflavone-free feed | Sintetic isoflavone  Genistein (isolated): 15 mg/Kg | Daily | 25 |
| Roberts et al., 2000  [26] | Gestation | Oral  (Food intake) | Isoflavone-free feed | (?)  Genistein (isolated): 5 mg/Kg | Daily | 130 |
| Delclos et al., 2001  [27] | Gestation | Oral  (Food intake) | Isoflavone-free feed | Toronto researc chemicals, Inc. ®  Genistein (isolated): 5, 25, 100, 250, 625 and 1250 mg/L | Daily | 50 |
| Robertson et al., 2002  [28] | Gestation | Oral  (Food intake) | Isoflavone-free feed | Soy meal 10%  Genistein/Daidzein (conjugated): 146 mg/Kg | Daily | 65 |
| Cline et al., 2004  [29] | 30 days | Oral  (Food intake) | Isoflavone-free feed | DuPont Protein Technologies®  Genistein/Daidzein (conjugated): 202.8 mg/Kg | Daily | 112 |
| Lee et al., 2004  [30] | 180 days | Oral  (Gavage) | Corn oil | Sigma Chemical®  Genistein (isolated): 2.5 mg/Kg | Daily | 35 |
| Mcvey et al., 2004  [31] | 50 days | Oral  (Food intake) | Isoflavone-free feed | Sigma Chemical®  Genistein/Daidzein/Glycitein (conjugated): 31.07, 36.10, 74.50, 235.80 and 1,046.60 mg/Kg | Daily | 360 |
| Faqi et al., 2004  [32] | 42 days | Oral  (Food intake) | Isoflavone-free feed | PTI G-2535® National Cancer Institute  Genistein/Daidzein/ glycitein (conjugated): 200 and 2000 mg/Kg | Daily | 365 |
| Jaroenporn et al., 2006  [33] | 56 days | Oral  (Food intake) | Destilled water | Isolated plant (Pueraria mirifica), northern Thailand  Genistein/Daidzein/Glycitein (conjugated): 10 and 100 mg/Kg | Daily | 56 |
| Assinder et al., 2007  [34] | 90 days | Oral  (Food intake) | Diet 86 | Diet RMH 3500 - Reliance Stockfoods®  Genistein/Daidzein/Glycitein (conjugated): 0.465 mg/Kg | Daily | 24 |
| Akingbemi et al., 2007  [35] | Gestation | Oral  (Food intake) | Casein | Tarlan-Heklad ®, Indianapolis, IN  Genistein/Daidzein/Glycitein (conjugated): 5, 50, 500 and 1000 mg/L | Daily | 21 |

**Table S3 (*Continued*).** Description of the main experimental characteristics of studies that evaluated the action of isoflavones on testicular function in murine models.

| **Study** | **Beginning of treatment** | **Route** | **Control** | **Treated**  **(Isoflavone-enriched diet)** | **Frequency** | **Duration (day)** |
| --- | --- | --- | --- | --- | --- | --- |
| Guan et al., 2008  [36] | 28 days | Oral  (Gavage) | Destilled water | Zhejiang Xinxin Bio-Tech®  Genistein/Daidzein (conjugated): 30, 150, 300 and 600 mg/Kg | Daily | 84 |
| Sherrill et al., 2010  [37] | Gestation | Oral  (Food intake) | Casein | Plant isolated (Soy) - Harlan-Teklad®  Genistein/Daidzein/ Glycitein (Conjugated): 5, 50, 1000 mg/L | Daily | 90 |
| Cederroth et al., 2010  [38] | Gestation | Oral  (Food intake) | Isoflavone-free feed | (?)  Genistein/Daidzein (conjugated): 150 and 190 mg/L | Daily | 180 |
| Piotrowska et al., 2011  [39] | Gestation | Oral  (Food intake) | Standard diet | Meno Stop®  Genistein/Daidzein (conjugated): 200 mg/Kg | Daily | 90 |
| Ekaluo et al., 2011  [40] | 90 days | Oral  (Food intake) | Commercial diet | Livestock Fram® (Soybean meal)  Genisteín/Daidzein (conjugated): 100, 200 and 300 mg/Kg | Daily | 60 |
| Modaresi et al., 2011  [41] | (?) | Oral  (Food intake) | Isoflavone-free feed | Plant isolated (Soy)  Genistein/Daidzein (conjugated): 20, 30, 50% in food | Daily | 63 |
| Pfaehler et al., 2012  [42] | Gestation | Oral  (Food intake) | Casein | Plant isolated (Soy) - Harlan-Teklad®  Genistein/Daidzein (Conjugated): 0.1, 5, 50, 1000 mg/L | Daily | 90 |
| Loutchanwoot et al., 2013  [43] | 90 days | Oral  (Gavage) | Olive oil | Changzhou Dahua Imp. and Exp. ®  Equol (isolated): 100 and 250 mg/Kg | Daily | 5 |
| Musameh et al., 2014  [44] | Gestation | Subcautaneous | Tween-80 | Indofine Chemical Com. ®  Genistein (isolated): 1, 10 and 100 mg/Kg | Daily | 21 |
| Loutchanwoot et al., 2014  [45] | 120 days | Oral  (Gavage) | Olive oil | Changzhou Dhua Imp. and Exp. ®  Equol (isolated): 100 and 250 mg/Kg | Daily | 5 |
| Meena et al., 2016  [46] | Gestation | Intraperitoneal | Dimethylsulfoxide | Chengdu Biopurify Phytochemicals®  Genistein (isolated): 2, 20 and 100 mg/Kg | Daily | 100 |
